# Obituary

**Published:** 1909-10-09

**Authors:** 


### Obituary.

Dr. Oglethorpe Wakelin Barratt, of Holloway Head, Birmingham, who died on July 9, aged 83, left estate of the gross value of £14,705, with net personalty £7,049.

Dr. Daniel John Cunningham, Professor of Anatomy in the University of Edinburgh, who died on June 23, aged 59, left personal estate valued at £17,846.

Surgeon-Colonel Ebenezer Robert Butler, I.M.S. (retired), whose death is announced in his seventy-seventh year, had held the posts of surgeon to the European General Hospital, superintendent of vaccination, and Presidency Surgeon at Bombay. He was medically educated at Guy's Hospital, and obtained the membership of the Royal College of Surgeons in 1853 and the M.D. degree of St. Andrews University in 1854. Surgeon-Colonel Butler formerly resided at South Kensington.

The death is announced of Dr. Edward Clapton, of Towercroft, Lee, S.E. He took his degree at London University in 1857, and became physician to St. Thomas's Hospital. He was the author of several works of ecclesiastical interest, and had retired from practice at the time of his death. Besides the M.D. degree of the University of London Dr. Clapton was the possessor of many Fellowships, including those of the Linnean and the Zoological Societies. Dr. Clapton's death recalls the recent presentation of a unique "specimen" which he made to the museum of the Royal College of Surgeons. It consisted of two branches and a bundle of twigs from the plane tree in the island of Cos under the shade of which Hippocrates is reputed to have taught the healing art.

The death is announced of Mr. William Lister, of Leeds. He was born at Birstall seventy-seven years ago, and was a district councillor and a member of the Education Sub-Committee of Oakwell Joint Hospital Board.

The death is announced at Berlin, in her forty-ninth year, of Dr. Agnes Hacker, who was one of the first German women to adopt the career of medicine as a profession, twenty years ago. She received her medical education at Zürich, and after taking her degree continued her studies at Vienna and Leipzig before taking up a private practice at Berlin. A year ago Dr. Hacker established a small hospital where women of all classes could receive surgical treatment at the hands of qualified medical practitioners of their own sex.

The death is announced of Dr. William Rivers Pollock, obstetric physician and lecturer on midwifery at the Westminster Hospital. Dr. Pollock, who was born in 1859, graduated M.B., B.C., at Cambridge in 1888. Three years later he obtained the Fellowship of the Royal College of Physicians and the M.D., Cambridge, in 1903. Devoting himself to the study of obstetrics, he became Examiner in Midwifery and Diseases of Women to the Conjoint Board, and Cambridge University and London University. He held, besides the Westminster Hospital appointments, the posts of senior physician to the Queen Charlotte Lying-in Hospital, physician to the Grosvenor Hospital for Women and Children, and professor of midwifery to the Army Medical College, Millbank.

The death took place on Monday, October 4, of Dr. Frederick Carter, of Billericay. He was a son of the late Dr. Carter, who had practised in Billericay for fifty years, and he himself had completed forty-six years' work when he retired. Among the posts held by Dr. Carter were those of Medical Officer of Health to the Billericay Union Sanitary District, and medical officer to the Billericay Workhouse, and to the Poplar Labour Colony at Laindon. When the announcement of his retirement was made, a subscription of £303 was made towards a public testimonial to him.

